# Effect of acupuncture on cognitive impairment induced by sleep deprivation in animal models: a preclinical systematic review and meta-analysis

**DOI:** 10.3389/fnagi.2025.1560032

**Published:** 2025-03-19

**Authors:** Chang Liu, Yutong Su, Yuen-ming Yau, Huize Lin, Yihao Chen, Weijian Fang, Nenggui Xu, Zhennan Wu

**Affiliations:** ^1^South China Research Center for Acupuncture and Moxibustion, Medical College of Acu-Moxi and Rehabilitation, Guangzhou University of Chinese Medicine, Guangzhou, China; ^2^Monash Neuromodulation Research Unit, Department of Physiotherapy, Faculty of Medicine, Nursing and Health Science, School of Primary and Allied Health Care, Monash University, Frankston, VIC, Australia

**Keywords:** acupuncture, sleep deprivation, cognitive impairment, meta-analysis, animal models

## Abstract

**Background:**

Sleep deprivation (SD) has been associated with cognitive deficits, mediated by mechanisms such as neuroinflammation and oxidative stress. Acupuncture, a core component of traditional Chinese medicine, has shown promise in mitigating SD-induced cognitive impairment. However, the effectiveness and underlying mechanisms of acupuncture need further validation through high-quality evidence. This study aims to evaluate the therapeutic effects and molecular mechanisms of acupuncture on cognitive impairment resulting from SD by conducting a systematic review and meta-analysis.

**Methods:**

This study comprehensively searched eight databases for randomized controlled trials (RCTs) that examine the effects of acupuncture on SD-induced cognitive impairment. Primary outcomes were assessed using the Morris Water Maze (MWM), including measures of escape latency and time spent in the target quadrant. Secondary outcomes focused on molecular markers such as brain-derived neurotrophic factor (BDNF), tropomyosin receptor kinase B (TrkB), and indicators of oxidative stress. The risk of bias was evaluated using the SYRCLE tool, and data were analyzed using R software. Standardized mean differences (MD) and 95% confidence intervals (CIs) were calculated.

**Results:**

Eight RCTs involving 222 rodents were analyzed. The findings indicate that acupuncture significantly improves cognitive performance in SD models, evidenced by increased platform crossings [MD = 1.67, 95% CI (1.42, 1.91)] and extended time in the target quadrant [MD = 8.54, 95% CI (6.35, 10.73)], along with reduced escape latency [MD = −8.33, 95% CI (−11.68, −4.99)]. Electroacupuncture (EA) was found to regulate the expression of BDNF and its receptor, TrkB, and to decrease oxidative stress markers such as malondialdehyde (MDA) while enhancing antioxidant activities, including those of superoxide dismutase (SOD). Manual acupuncture (MA) influenced apoptosis markers by decreasing Bax and increasing Bcl-2 expression. Despite these positive findings, the studies exhibited heterogeneity in intervention methods and variability in acupuncture techniques.

**Conclusion:**

This study preliminarily confirms that acupuncture, specifically electroacupuncture, and manual acupuncture, can effectively alleviate cognitive impairment caused by sleep deprivation. The benefits are observed through modulation of BDNF–TrkB signaling, reduction in oxidative stress, and regulation of apoptosis. Although the current evidence is derived from animal studies, it suggests potential applications in human clinical trials to explore the viability of acupuncture for treating cognitive impairment related to SD.

**Systematic review registration:**

CRD42024627285, https://www.crd.york.ac.uk/PROSPERO/.

## 1 Introduction

Cognitive impairment induced by sleep deprivation (SD) has emerged as a significant public health concern, particularly among the elderly and individuals with chronic sleep disorders, such as shift workers or patients with obstructive sleep apnea (Wennberg et al., 2017; Shi et al., 2018; Dubois et al., 2021). Increasing evidence suggests that SD disrupts neuroinflammatory homeostasis, exacerbates oxidative stress, and accelerates the deposition of *β*-amyloid (Aβ), a hallmark of Alzheimer’s disease (AD) (Xue et al., 2019; Chen and Zhong, 2014). A bidirectional relationship exists between sleep and AD pathology (Ju et al., 2014); insufficient sleep can lead to Aβ accumulation, while the formation of amyloid plaques may disrupt sleep quality by damaging brain regions that regulate sleep (Ju et al., 2013). In APP/PS1 transgenic mice, significant Aβ deposition coincides with disturbed sleep patterns starting at 6 months, worsening by 9 months (Roh et al., 2012). Research indicates that Aβ is cleared significantly faster during sleep than while awake, and SD may impair this self-clearing mechanism (Xie et al., 2013; Duarte et al., 2020; Holth et al., 2019). Despite existing pharmacological treatments like cholinesterase inhibitors and glutamate antagonists, no FDA-approved medications effectively address SD-related cognitive deficits, and current therapies often have considerable side effects or limited efficacy (Wang et al., 2021). This gap underscores the urgent need for alternative interventions.

Acupuncture, a core component of traditional Chinese medicine, has shown promising clinical efficacy in managing cognitive and sleep disorders (Lu et al., 2022). Animal studies have suggested that acupuncture may enhance cognitive function and sleep quality through various mechanisms, including neuroprotection and anti-inflammatory effects (Xiao et al., 2018; Ji et al., 2021; Wu et al., 2023). However, these results are scattered across small-scale animal studies with methodological variability, such as differing acupuncture parameters and outcome measures. This inconsistency has impeded clinical translation. No previous reviews have specifically addressed SD-induced cognitive impairment in animal models. This meta-analysis fills that gap by rigorously evaluating the efficacy of acupuncture in preclinical SD models, elucidating its molecular mechanisms, and laying the groundwork for future clinical trials. By synthesizing high-quality evidence, this study aims to bridge the translational gap between animal research and human clinical applications, highlighting acupuncture’s potential as a safe, non-pharmacological therapy for cognitive disorders.

## 2 Methods

### 2.1 Study registration

This study adheres to the Preferred Reporting Items for Systematic Reviews and Meta-Analyses (PRISMA) guidelines specific to aging neuroscience and follows the PRISMA checklist. The protocol was registered with the International Prospective Register of Systematic Reviews (PROSPERO) on December 17, 2024, under registration number CRD42024627285.

### 2.2 Literature search

An extensive literature search was conducted by two researchers across eight databases and completed by December 7, 2024. This included four English-language databases—Cochrane Library, PubMed, Embase, and Web of Science—and four Chinese-language databases—China Biology Medicine, VIP, Wanfang Data, and CNKI. Publications from all countries, in both Chinese and English, were considered, encompassing various article types. The focus was on a meta-analysis of acupuncture treatment for cognitive impairment caused by sleep deprivation. Keywords used included “sleep deprivation,” “cognitive impairment,” “acupuncture,” “animal experiments,” and “meta-analysis.” Details of the search strategy are provided in Supplementary material 1.

### 2.3 Study selection

The following inclusion criteria were applied during the study selection process: 1. Randomized controlled trials (RCTs) utilizing animal models of sleep deprivation; 2. Studies that effectively established sleep deprivation animal models using diverse methodologies, with no limitations on species, sex, or age; 3. Interventions employing manual acupuncture, electroacupuncture, or a combination of both with other therapies. There were no limitations on acupuncture points, techniques, angles, treatment duration, courses, or electroacupuncture parameters, and no restrictions on the form of combined therapies; 4. Studies reporting outcomes based on the number of platform crossings, escape latency, and time spent in the target quadrant in the Morris Water Maze (MWM); 5. No restrictions on region and language. Exclusion criteria included: 1. Animal studies that were not RCTs; 2. Studies where the treatment group did not use any form of manual or electroacupuncture; 3. Duplicate publications or studies with redundant data; 4. Studies that did not include MWM as an outcome measure; 5. Studies with incomplete data, conference abstracts, or editorial commentaries; 6. Other studies that, upon manual screening, failed to meet the inclusion criteria.

### 2.4 Data extraction

After completing the search, the retrieved documents were imported into EndNote X9 software. Following the removal of 37 duplicate articles, two reviewers independently screened the titles and abstracts of 155 articles, eliminating 104 studies that did not meet the inclusion criteria. The full texts of the remaining studies were then reviewed to confirm eligibility, ultimately including eight studies that met all criteria. The data extraction process was carried out by two researchers, who collected details such as the first author, publication year, sample size, randomization methods, and grouping details; specifics about the subjects, such as sex, age, and duration of disease; intervention details, including the number of treatments, acupuncture points used, and electroacupuncture parameters; and outcome data. In cases of disagreement, a third reviewer was consulted to make the final decision.

### 2.5 Risk of bias assessment

Two researchers, Yihao Chen, and Weijian Fang, independently assessed the risk of bias using the Systematic Review Centre for Laboratory Animal Experimentation (SYRCLE) Risk of Bias Tool (RoBT) (Hooijmans et al., 2014). The RoBT evaluates bias across ten domains: 1. Sequence generation; 2. Baseline characteristics; 3. Allocation concealment; 4. Random housing; 5. Blinding of caregivers and researchers; 6. Random outcome assessment; 7. Blinding of outcome assessors; 8. Incomplete outcome data; 9. Selective outcome reporting; and 10. Other sources of bias. Discrepancies in assessments were resolved through discussion between the two researchers. If necessary, a third researcher, Huize Lin, was consulted for arbitration.

### 2.6 Data analysis

Data analysis was performed using R software, version 4.3. All outcomes were treated as continuous variables. When results were reported using different measures or units across studies, the mean difference (MD) was used as the effect size index. Confidence intervals (CI) were set at 95%, with a *p*-value less than 0.05 considered statistically significant. Heterogeneity was assessed using the Q test and I^2^ statistic. A fixed-effect model was applied when I^2^ ≤ 50%, and a random-effects model was used otherwise. Sensitivity analyses were conducted to test the stability and reliability of the results.

## 3 Results

### 3.1 Literature screening process

**Figure 1:**
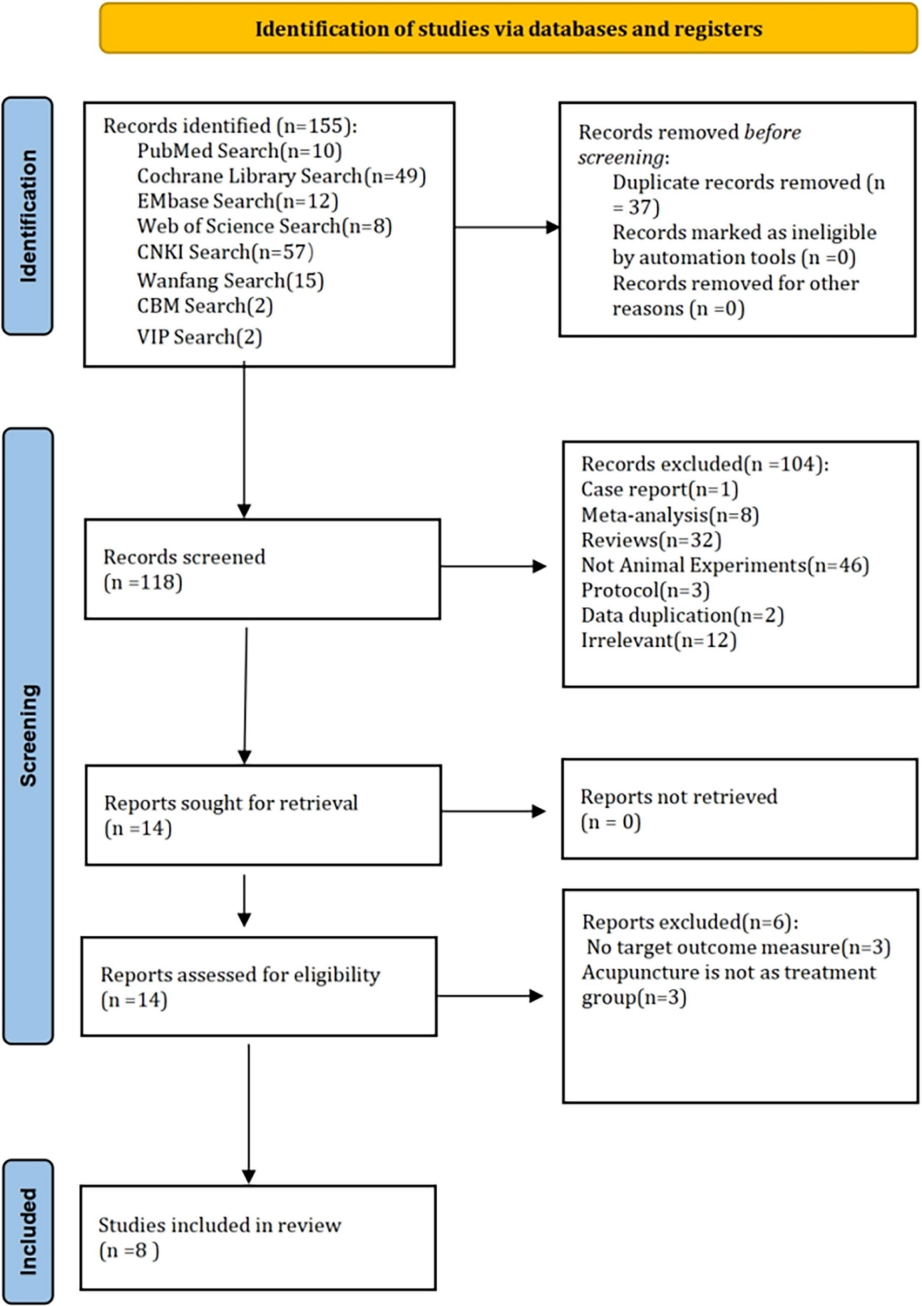
Literature screening process.

Using a predefined search strategy, two independent reviewers (Yutong Su and Yuen-ming Yau) retrieved a total of 155 articles from multiple databases (PubMed, Cochrane Library, Embase, Web of Science, CNKI, Wanfang, CBM, and VIP). After removing 37 duplicates, 118 articles advanced to the initial screening phase. During this phase, titles and abstracts were reviewed, and 104 studies were excluded for the following reasons: 1. Case reports (1 article); 2. Meta-analyses (8 articles); 3. Reviews (32 articles); 4. Non-animal experimental studies (46 articles); 5. Protocol articles (3 articles); 6. Duplicate data (2 articles); 7. Irrelevant topics (12 articles). The remaining 14 articles proceeded to a full-text review, all of which were successfully retrieved with no reports missing. During further eligibility assessment, six articles were excluded because: 1. They failed to report target outcome measures (3 articles); 2. Acupuncture was not used as the intervention in the treatment group (3 articles). Ultimately, eight articles were selected for inclusion in the systematic review and meta-analysis (Figure 1).

### 3.2 Characteristics of included studies

This review encompasses eight studies involving a total of 222 rodents, split evenly between treatment and control groups. The studies are comprised of four English-language articles (Hooijmans et al., 2014; Wu et al., 2021; Liu, 2023; Qiao et al., 2023) and four Chinese-language articles (Zhao et al., 2021; Zhang et al., 2022; Hao et al., 2022; Yang, 2019) published between 2019 and 2023 (Table 1). In the literature reviewed, GV20 was selected as an acupoint in seven studies, while one study did not use GV20(Wu et al., 2021). Three studies (Liu, 2023; Qiao et al., 2023; Zhao et al., 2021) employed both GV20 and SP6, but the selection of other acupoints varied across the studies, leading to a lack of consistency. Two studies used manual acupuncture (MA) (Zhao et al., 2021; Zhang et al., 2022), while the remaining six used electroacupuncture (EA). Among the EA studies, the selected waveforms included continuous wave (2 studies) (Liu, 2023; Hao et al., 2022), sparse-dense wave (3 studies) (Wu et al., 2021; Qiao et al., 2023; Yang, 2019), and biphasic square pulse (1 study) (Xu et al., 2020). EA frequencies ranged from 2 Hz to 100 Hz. Specifically, two studies used 2 Hz (Wu et al., 2021; Hao et al., 2022), one study used a biphasic square pulse involving 2 Hz and 15 Hz (Qiao et al., 2023), Xu et al. (2020) selected 100 Hz, Liu (2023) used 20 Hz, and Yang (2019) used 4 Hz. Regarding EA intensity, 1 mA was the most common setting (three studies) (Liu, 2023; Qiao et al., 2023; Hao et al., 2022), followed by 2 mA (two studies) (Yang, 2019; Xu et al., 2020), with Wu et al. (2021) using 0.5 mA. Notably, both MA studies employed a twirling manipulation with bi-directional needle rotation at a speed of 180° per second during treatment. The studies that used rats included six studies with Sprague–Dawley rats, one with Wistar rats (Wu et al., 2021), and one with mice (Xu et al., 2020). Sleep deprivation methods included the improved multiplatform water environment method (six studies) (Wu et al., 2021; Zhao et al., 2021; Zhang et al., 2022; Hao et al., 2022; Yang, 2019; Xu et al., 2020) and intraperitoneal injection of PCPA (two studies) (Liu, 2023; Qiao et al., 2023). All eight studies used the MWM to assess cognitive function. However, the outcome measures varied: three articles (Zhao et al., 2021; Hao et al., 2022; Yang, 2019) measured platform crossings, target quadrant time, and escape latency. Xu et al. (2020) measured platform crossings and target quadrant time. Liu (2023) only measured platform crossings. And three articles (Wu et al., 2021; Qiao et al., 2023; Zhao et al., 2021) focused solely on escape latency. All studies examined hippocampal tissue. Wu et al. (2021), Qiao et al. (2023), and Xu et al. (2020) measured BDNF and TrkB, while the other studies investigated various molecular markers.

**Table 1:** Characteristics of included studies.

		Animal models							Intervention (treatment group)				Intervention (control group)	Specimen	Cytokines and inflammatory mediators	Behavioral tests
		Species	Sex	Age	Weight (g)	n=treatment group/control group	Method of establishing model	Sample size	Type	Acupoints	Treatment time and duration	Electroacupuncture parameters				
1	Hao et al. (2022)	Sprague Dawley rats	Male	2 months	220–250	12/12	Improved multiplatform water environment method	24	EA	GV20, GV14	15 min, once daily for 7 days	Continuous wave, 1 mA and 2 Hz	/	Hippocampus	a, b, c, d	①, ②, ③
2	Xu et al. (2020)	BALB/c mice	Male	2–3 month	/	34/34	Improved multiplatform water environment method	68	EA	GV 20	30 min, once daily for 3 days	Biphasic square pulse, 2 mA and 100 Hz	/	Hippocampus	e, f	②, ③
3	Wu et al. (2021)	Wistar rats	/	/	180–200	8/8	Improved multiplatform water environment method	16	EA	GB20	20 min, once daily for 14 days	Sparse Wave, 0.5 mA, 2 Hz	/	Hippocampus	e, f, g	①
4	Liu (2023)	Sprague Dawley rats	Male	/	200 ± 2	10/10	Intraperitoneal injection of PCPA	20	EA	GV20, SP6	30 min, once daily for 7 days	1 mA, 20 Hz, continuous wave	/	Hippocampus	h, j	①
5	Qiao et al. (2023)	Sprague Dawley rats	Male	2 months	220 ±20	11/11	Intraperitoneal injection of PCPA	22	EA	GV20, HT7, SP6	30 min daily for 4 days	Alternating frequency of 2/15 Hz and an intensity of 1 mA	/	Hippocampus	e, f, h, i, k, l	①, ③
6	Yang (2019)	Sprague Dawley rats	/	/	200–230	10/10	Improved multiplatform water environment method	20	EA	GV20, DU24	30 min daily for 5 days	Dense-Sparse Wave, 4 Hz, 2 mA	/	Hippocampus	m, n, o, p	①, ②, ③
7	Zhang et al. (2022)	Sprague Dawley rats	Male	2 months	210–230	12/12	Improved multiplatform water environment method	24	MA	DU26, GV20, PC6, CV4	For 5 days	PC6 inserting and reducing method by twirling the needle for 30 s. The reinforcing method of GV 20 was twirled for 30 s, and the reducing method of CV 4 was twirled for 30 s	Two non-acupoints were fixed under the ribs on both sides, and the twirling manipulation of flat reinforcing and reducing was used for 20 s	Hippocampus	p, q, r, s	①
8	Zhao et al. (2021)	Sprague Dawley rats	Male	/	200 ± 20	14/14	Improved multiplatform water environment method	28	MA	GV20, HT7, SP6, GV29	20 min daily for 3 days every weeks last 6 weeks	During treatment, twirling manipulation was applied every 5 min and lasted 15 s each time. Each needle was rotated bi-directionally within 180° in one second	Hallow puncture at non-acupoints near the real acupoints	Hippocampus, prefrontal cortex	t, u, v, w, x	①, ③

①: Escape latency; ②: the duration in platform quadrant; ③: platform crossing number; a: miR-132-3p; b: p250GAP; c: Cdc42; d: Rac1; e: BDNF; f: TrkB; g: SYN; h: Ras; i: c-raf; j: ERK1/2; k:PKA-Cβ; l: p-CREBSA; m: NLRP3; n: Caspase-1; o: IL-1β; p: β-actin; q: DDAH; r: NO; s: ADMA; t: SOD; u: MDA; v: GSH-P; w: Bax; x: Bcl-2; y: GAPDH; EA: Electroacupuncture; SA: Sham Acupuncture; MA: Manual Acupuncture.

### 3.3 Risk of bias

**Figure 2:**
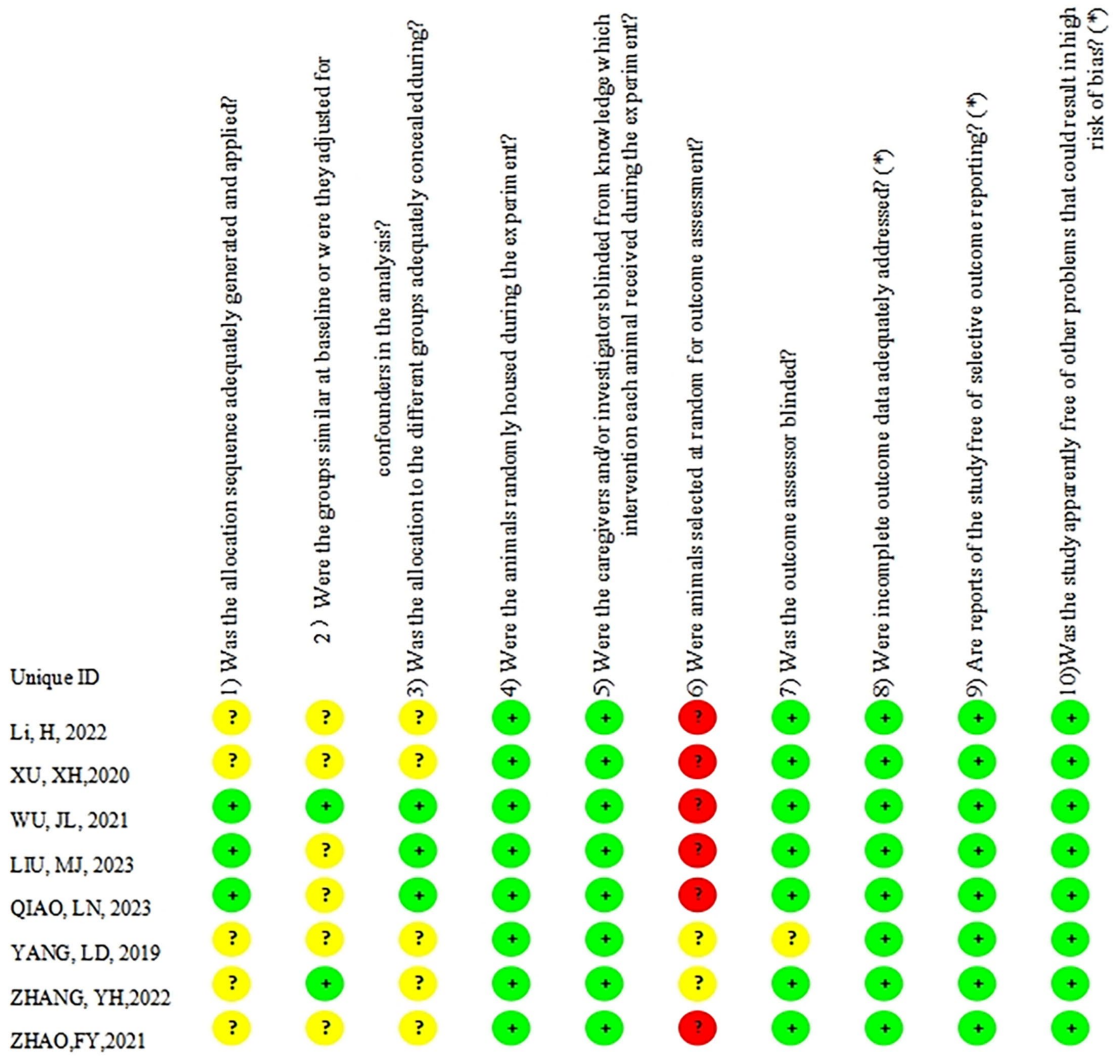
Risk of bias assessment for included studies.

Assessment Using the SYRCLE Risk of Bias Tool for Animal Studies (Figure 2). The bias risk of the eight included studies was assessed as follows: Three studies (Wu et al., 2021; Liu, 2023; Qiao et al., 2023) explicitly reported sequence generation and were rated as “low risk of bias.” The remaining five studies (Zhao et al., 2021; Zhang et al., 2022; Hao et al., 2022; Yang, 2019; Xu et al., 2020) did not specify sequence generation and thus were rated as “unclear risk.” For baseline characteristics (Item 2), two studies (Wu et al., 2021; Zhang et al., 2022) reported balanced baselines. They were rated “low risk,” while the other six studies (Liu, 2023; Qiao et al., 2023; Zhao et al., 2021; Hao et al., 2022; Yang, 2019; Xu et al., 2020) did not specify baseline balance and were thus rated “unclear risk.” Allocation concealment (Item 3) was clearly reported in three studies (Wu et al., 2021; Liu, 2023; Qiao et al., 2023), rated as “low risk,” and rated as “unclear risk” in five studies (Zhao et al., 2021; Zhang et al., 2022; Hao et al., 2022; Yang, 2019; Xu et al., 2020). Random housing (Item 4): All eight studies reported random housing and were rated as “low risk.” Blinding of caregivers (Item 5): All eight studies reported caregiver blinding, also rated as “low risk.” Blinding of outcome assessors (Item 6): Six studies (Wu et al., 2021; Liu, 2023; Qiao et al., 2023; Zhao et al., 2021; Hao et al., 2022; Xu et al., 2020) did not use blinding for outcome assessors, rated as “high risk,” while two studies (Zhang et al., 2022; Yang, 2019) were rated as “unclear risk.” Incomplete outcome data (Item 7): Seven studies reported complete data, rated as “low risk,” while one study (Yang, 2019) was rated “unclear risk.” Selective outcome reporting (Item 8): All eight studies showed no selective reporting and were rated as “low risk.” Random selection of animals for outcome assessment (Item 9): All studies used random selection, rated as “low risk.” Other sources of bias (Item 10): No other biases were identified and were rated as “low risk.” The overall assessment indicated minimal risk of bias in randomization, data completeness, and outcome reporting, yet higher risks were observed in sequence generation, allocation concealment, baseline balance, and blinding of outcome assessment.

### 3.4 Behavioral test results

After sensitivity analysis, I^2^ = 0% for the number of platform crossings after removing Liu MJ, and I^2^ = 27% for platform quadrant dwell time after removing Xu XH; therefore, a fixed effects model was applied in both instances. For escape latency, I^2^ increased to 71% after removing Qiao LN, necessitating the use of a random-effects model (Supplementary Figure S2). Data from three to four studies involving 68 to 92 rodents comparing acupuncture with control groups demonstrated: 1. Platform Crossing Number: The treatment group exhibited a significantly higher number of crossings compared to the control group (MD = 1.67, 95% CI = 1.42–1.91); 2. Duration in Target Quadrant: The treatment group spent significantly more time in the target quadrant (MD = 8.54, 95% CI = 6.35–10.73); 3. Escape Latency: The treatment group demonstrated significantly reduced escape latency (MD = −8.33, 95% CI = −11.68 to −4.99). Additionally, a subgroup analysis of EA vs. MA based on these three outcomes revealed the following: 1. For the Platform Crossing Number, the EA group included two studies, and the MA group one study, with I^2^ = 0% and MD = 1.76, 95% CI = 1.40–2.13 for the EA group, and MD = 1.58, 95% CI = 1.25–1.91 for the MA group; 2. For the Duration in Target Quadrant, the EA group consisted of two studies and the MA group one study, with I^2^ = 0%, MD = 9.88, 95% CI = 6.99–12.76 for the EA group, and MD = 6.72, 95% CI = 3.36–10.08 for the MA group; 3. For Escape Latency, both the EA and MA groups contained two studies each, with I^2^ = 0%, MD = −8.44, 95% CI = −11.49 to −5.39 for the EA group; and I^2^ = 89%, MD = −6.39, 95% CI = −8.20 to −4.52 for the MA group.

**Figure 3:**
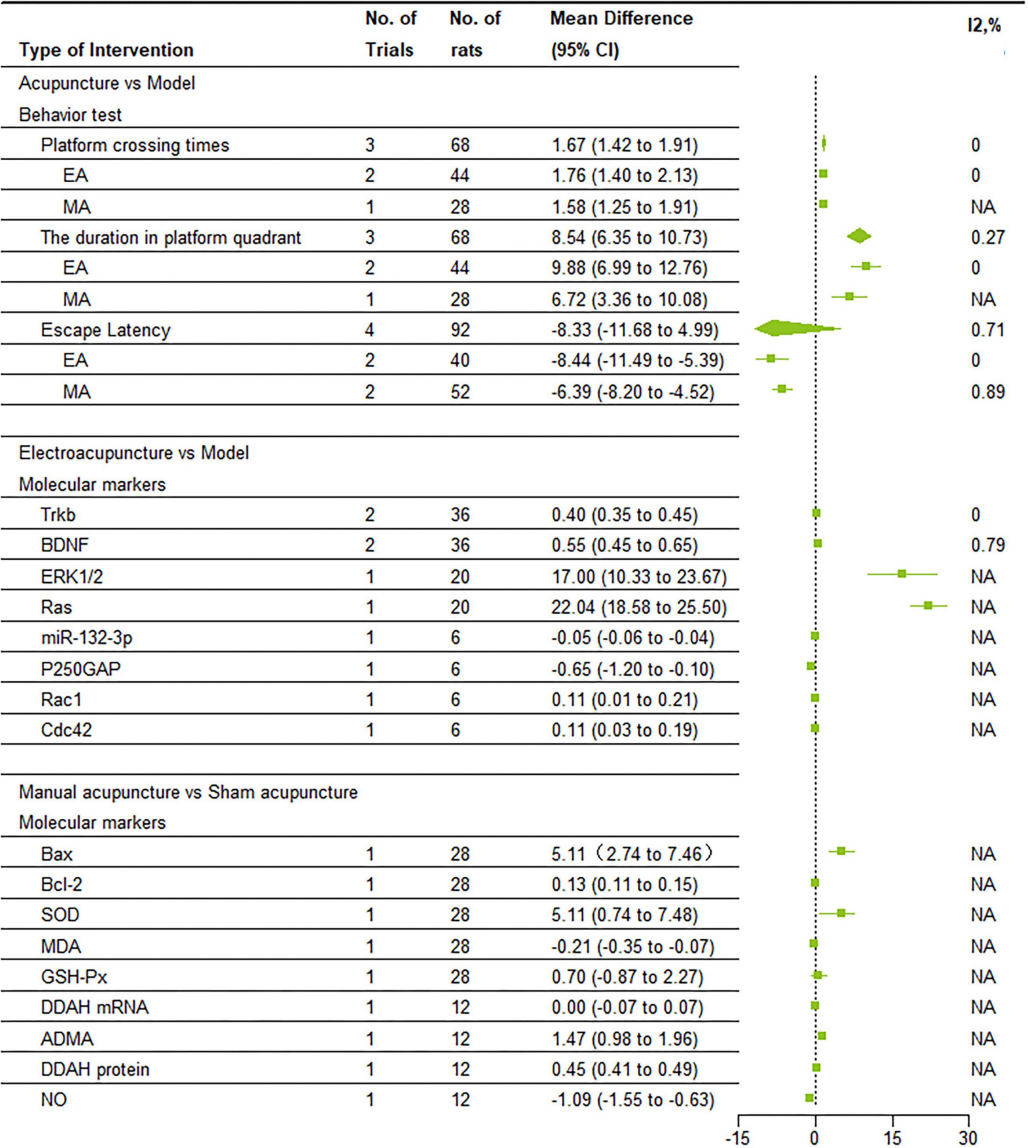
Forest plots of result of meta analysis.

These findings indicate that acupuncture significantly enhances spatial learning and memory, as evidenced by increased time in the target quadrant, decreased escape latency, and increased platform crossings. The treatment results are notably better with EA (Figure 3).

### 3.5 Molecular marker changes after EA

After sensitivity analysis, the results for TrkB and BDNF after removing Qiao LN were I^2^ = 0% and I^2^ = 79%, respectively. Consequently, a fixed-effect model was used for TrkB, while a random-effects model was applied for BDNF (Supplementary Figure S2). Data from 1 to 2 studies, involving 6–36 rodents, showed the following results: Firstly, EA significantly increased the expression of TrkB and BDNF (MD = 0.40, 95%CI = 0.35–0.45; MD = 0.55, 95%CI = 0.45–0.65). Secondly, electroacupuncture significantly increased the expression of ERK1/2 and Ras (MD = 17.00, 95%CI = 10.33–23.67; MD = 22.04, 95%CI = 18.58–25.50). The third EA decreased the expression of miR-132-3p and P250GAP (MD = −0.05, 95%CI = −0.06 to −0.04; MD = −0.65, 95%CI = −1.20 to −0.10). Finally, EA significantly increased the expression of Rac1 and Cdc42 (MD = 0.11, 95%CI = 0.01–0.21; MD = 0.11, 95%CI = 0.03–0.19).

These findings indicate that EA may improve cognitive function by modulating signaling pathways involving TrkB, BDNF, ERK1/2, and Ras (Figure 3).

### 3.6 Molecular marker changes after MA

Data from one study, involving 12–28 rodents, showed the following changes: 1. Apoptosis-related Proteins: MA reduced Bax expression (MD = 0.13, 95% CI = 0.11–0.15) and increased Bcl-2 expression; 2. Antioxidant Activity: MA increased SOD activity (MD = 5.11, 95% CI = 0.74–7.48) and reduced MDA levels (MD = −0.21, 95% CI = −0.35 to −0.07); 3. Other Molecular Markers: MA increased the expression of GSH-Pxs (MD = 0.70, 95% CI = 0.87–2.27), ADMA (MD = 1.47, 95% CI = 0.98–1.96), and DDAH protein (MD = 0.45, 95% CI = 0.41–0.49), while reducing NO levels (MD = −1.09, 95% CI = −1.55 to −0.63).

These results suggest that MA mitigates oxidative damage and promotes neuroprotection through the regulation of apoptotic and antioxidant molecules (Figure 3).

## 4 Discussion

There is a broad correlation between sleep deprivation (SD) and cognitive impairment, featuring a bidirectional relationship. While some scholars have explored related areas in recent years, to the best of my knowledge, no study has systematically examined the effects of acupuncture on SD-induced cognitive impairment. This study provides preliminary evidence that both electroacupuncture (EA) and manual acupuncture (MA) significantly improve cognitive deficits induced by SD in animal models. Pooled results from eight animal experiments demonstrate that acupuncture enhances spatial learning and memory, as evidenced by increased platform crossings in the Morris Water Maze (MWM), longer durations in the target quadrant, and shorter escape latencies. Interestingly, in the subgroup analysis of the three outcome measures of the water maze, we observed extremely high homogeneity (I^2^ = 0) in the EA group and extremely high heterogeneity in the MA group. This prompted us to review the intervention parameter Settings of EA and MA in related studies. We noted that the EA group showed less variability, probably because similar parameter Settings (frequencies between 2 and 4 Hz and current intensities between 0.5 mA and 2 mA) were maintained across studies. In contrast, the MA group showed a completely different acupuncture pattern. In addition, the heterogeneity was significantly higher when the sensitivity analysis of the platform crossing number included the study by Liu Meijie before the subgroup analysis. This prompted us to review its EA parameters; its frequency was 20 Hz, which was significantly different from other EA studies. This suggests that the main reason for the heterogeneity of studies with similar criteria may be related to the acupuncture method. Due to the diversity of MA operation techniques and the level of operators, it often leads to significant treatment variability. In contrast, due to its controllable parameters and less dependence on the skill of the operator, EA allows a more consistent therapeutic effect within a standardized range. These behavioral improvements were accompanied by molecular changes, including upregulation of BDNF–TrkB signaling, reduction of oxidative stress markers such as MDA, and modulation of apoptosis-related proteins such as Bax/Bcl-2. These findings are consistent with existing preclinical studies and suggest that acupuncture can potentially alleviate cognitive deficits associated with SD through multiple targeted mechanisms.

Translating these results to cognitive impairment associated with sleep deprivation (SD) in humans requires rigorous clinical validation. While our findings are derived from animal models, they have some implications. Clinically, SD is prevalent among the elderly and in neurodegenerative diseases such as Alzheimer’s Disease (AD). Acupuncture has long been used to treat sleep disorders and cognitive decline in humans, and some studies have reported improvements in sleep quality and memory in elderly populations and patients with mild cognitive impairment (MCI). For instance, a randomized trial demonstrated that electroacupuncture (EA) improved cognitive function in patients with cerebral infarction-related insomnia, aligning with our preclinical findings. Additionally, some biochemical indices regulated by acupuncture treatment have been somewhat validated in human experiments. For example, it has been reported that elevated levels of brain-derived neurotrophic factor (BDNF) are significantly and positively correlated with memory improvement. Clinical studies have shown that acupuncture can effectively elevate patients’ serum BDNF levels (Zuppa et al., 2015; Sun et al., 2016). Furthermore, acupuncture has been shown to significantly increase serum BDNF levels in patients with insomnia (Yan et al., 2023; Liou et al., 2021). Consistent with the results from animal studies, EA can improve cognitive function by modulating neuroinflammation-related products. For instance, EA has been shown to improve cognitive function in patients with mild traumatic brain injury by decreasing the expression level of MDA (Liou et al., 2021). Similarly, acupuncture has been observed to reduce superoxide dismutase (SOD) expression in patients with vascular dementia, thereby improving their clinical symptoms (Kim et al., 2009). These pieces of evidence demonstrate that acupuncture can effectively regulate sleep and cognitive functions in patients and reinforce that acupuncture treatments are often characterized by their action on multiple targets.

However, translating these results to humans requires addressing critical gaps. First, standardized acupuncture protocols, such as acupoint selection and stimulation parameters, must be established to ensure reproducibility. Among these, EA is most suited for standardization due to its quantifiable parameters. Nevertheless, due to many influencing factors such as frequency, current intensity, and the selection of acupuncture points, establishing standardization remains a significant challenge and will be an important focus of future acupuncture research. The results of the current study suggest that low-frequency EA stimulation may be a major direction for future research. Second, long-term clinical studies are necessary to evaluate the sustainability and safety of these treatments. Third, integrating neuroimaging and biomarker assessments (e.g., plasma BDNF, oxidative stress markers) in future trials could help bridge the gap between preclinical and clinical findings. While animal studies provide a robust foundation for understanding acupuncture’s mechanisms, its clinical efficacy and feasibility require further validation. Future research should integrate functional brain imaging and neurobiological markers to elucidate the specific effects of acupuncture in humans.

Although the specific mechanisms by which SD affects cognitive function remain unclear, existing evidence suggests that factors such as amyloid-beta (Aβ), tau protein, and neuroinflammation significantly influence the progression of cognitive impairment and dementia (Chan et al., 2011). Acupuncture, as an integral part of traditional Chinese medicine, holds particular promise in treating cognitive and sleep-related disorders (Wang et al., 2021). Increasing evidence also indicates that acupuncture has significant therapeutic effects in modulating AD-like pathogens to improve neuroinflammation and regulate sleep (Zhang et al., 2022; Hao et al., 2022; Yang, 2019).

The cognitive improvements observed may be attributed to the modulation of key pathways by acupuncture. For instance, three animal experiments in this study demonstrated that electroacupuncture upregulated the expression of BDNF and TrkB, which are crucial for synaptic plasticity and neuronal survival. Given that the BDNF–TrkB signaling pathway is closely associated with hippocampus-dependent memory, this finding aligns with the improved performance in the MWM. Additionally, the reduction of oxidative stress markers such as malondialdehyde (MDA) and the enhancement of antioxidant activity (superoxide dismutase, SOD) further underscore the role of acupuncture in mitigating SD-induced neuronal damage. Similarly, manual acupuncture (MA) exhibited anti-apoptotic effects by modulating the Bax/Bcl-2 ratio, potentially safeguarding the integrity of the hippocampus. These mechanisms may act synergistically, with Ras and CREB serving as potential convergence points for downstream signaling.

Previous studies have shown that BDNF, through TrkB activation, promotes neuronal survival and synaptic plasticity and regulates AD pathology (Numakawa and Odaka, 2021). This pathway ultimately activates CREB, a crucial transcription factor. ERK, a major downstream protein activated by the BDNF–TrkB interaction, is essential for upregulating pre- and postsynaptic proteins, thereby facilitating synaptic plasticity (Kumamaru et al., 2008). Furthermore, BDNF binding to the TrkB receptor triggers TrkB phosphorylation, enhancing the formation of the Shc/Grb2/SOS complex, which leads to the activation of Ras and subsequently the Ras/Raf-1/MEK/ERK pathway (Chan et al., 2011). These findings also support our efforts to link SD-induced cognitive impairment with PKA/CREB and BDNF/TrkB signaling pathways and neuroinflammation across other research fields.

The hippocampus, a critical region for learning and memory, is particularly vulnerable to sleep deprivation damage, leading to cognitive impairments. Long-term potentiation (LTP), a cellular mechanism for synaptic plasticity, is mediated by molecules such as CaMKII and phosphorylated CREB. LTP, characterized by sustained enhancement of excitatory synaptic efficacy, is considered foundational for learning and memory (Duffy et al., 2001). BDNF, by binding to TrkB, promotes the formation and maintenance of LTP, further aiding in synaptic repair and cognitive improvement (Lu et al., 2013). Additionally, cAMP-dependent protein kinase (PKA) is crucial for hippocampus-dependent LTP and cognitive processes (Duffy et al., 2001). Once activated, the catalytic subunit of PKA (PKA-cβ) translocates to the nucleus, where it phosphorylates CREB, promoting the synthesis of proteins required for late-phase LTP and long-term memory. A reduction in phosphorylated CREB impairs LTP, suppressing the expression of critical target genes such as BDNF (Zagaar et al., 2016). Conversely, BDNF has been shown to regulate CREB activity, thus forming a positive feedback loop. The studies included in this research demonstrated that acupuncture activates the PKA/CREB and BDNF/TrkB signaling pathways, thereby mitigating cognitive impairments caused by sleep disorders (Wu et al., 2021; Qiao et al., 2023; Xu et al., 2020).

Neuroinflammation is considered one of the critical mechanisms linking sleep disorders to neurodegenerative diseases (Shi et al., 2018; Tobaldini et al., 2017). Notably, hippocampal inflammation is strongly associated with cognitive impairments. ADMA competitively inhibits nitric oxide synthase (NOS), which reduces nitric oxide (NO) production. NO plays a role in vasodilation and is closely related to conditions such as atherosclerosis, vascular spasms, and cognitive impairments associated with sleep apnea syndromes (Osorio-Yáñez et al., 2017; Hu et al., 2020). Downregulation of DDAH leads to ADMA accumulation, which in turn impedes NO production (MacAllister et al., 1996). Through nitrification, NO exerts a negative feedback loop on DDAH activity. The NOS/NO pathway is intricately linked to sleep regulation, and aberrant expressions of NO and inducible NOS (iNOS) increase with age, contributing to neurodegeneration and sleep disorders not attributable to physiological causes (Chen et al., 2003; Cui et al., 2015; Cespuglio et al., 2012). Animal studies have shown that the suppression of ROS and MDA levels in AD mice leads to increased expression of Ras, p-Raf, p-MEK, and p-ERK proteins, while enhancing SOD activity, consequently reducing Aβ and tau protein levels. Additionally, the included studies have demonstrated that acupuncture reduces NO and Bax expression, increases Bcl-2, MDA, GSH-Px, ADMA, Ras, and ERK1/2 expression, and improves cognitive function in sleep disorder-induced cognitive impairment models (Wang et al., 2024). This effect has been shown to reduce Bax (a pro-apoptotic protein) expression and increase Bcl-2 (an anti-apoptotic protein) levels, thereby protecting neurons (Deng et al., 2022). Bcl-2 regulates mitochondrial membrane permeability, preventing cytochrome C release and caspase activation, thereby reducing the release of inflammatory factors. Furthermore, the study investigated the role of miR-132 in synaptic plasticity, underscoring its importance in the nervous system and its dysregulation in various neurological diseases, including neurodegenerative disorders. It demonstrated that miR-132 enhances activity-dependent synaptic plasticity by inhibiting p250GAP expression and activating the Rac1-PAK-actin remodeling pathway (Vo et al., 2010). The studies included in this research similarly corroborate that acupuncture increases miR-132 expression, decreases p250GAP, and upregulates Rac1 expression, thereby improving cognitive function in a sleep disorder-induced cognitive impairment model.

**Figure 4:**
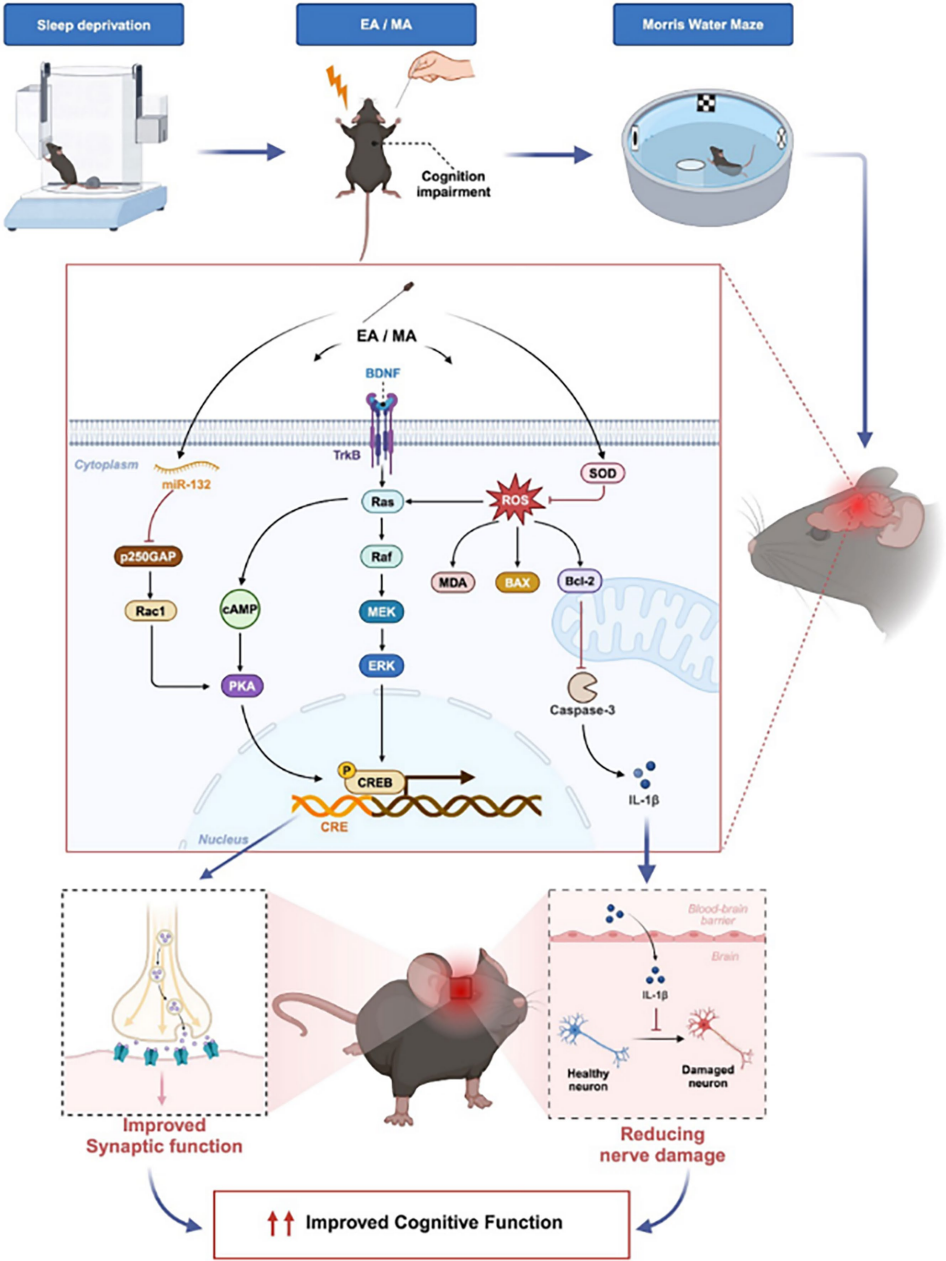
Potential mechanisms of acupuncture in the treatment of cognitive impairment caused by sleep deprivation.

Based on the results of our study and those of related scholars, we found that acupuncture can improve cognitive deficits that may result from sleep disorders through multiple pathways, as illustrated in Figure 4. 1. BDNF/TrkB/SOS/Ras/Raf/MEK/ERK/CREB pathway; 2. NOS/NO/ROS/MDA/SOD/Ras pathway; 3. miR-132/p250GAP/Rac1-PAK pathway. In these pathways, Ras is considered a key target that integrates multiple signaling routes to enhance synaptic function and cognition. CREB plays a critical role as a final effector in both synaptic function and cognitive improvement. Neuroinflammation is another target of acupuncture treatment, being a significant cause of cognitive impairment. The mechanisms may include the activation of SOD and the inhibition of ROS production by acupuncture, thereby reducing oxidative stress and inflammation. Although the evidence supporting the use of acupuncture for the treatment of cognitive impairment due to sleep disorders is still limited, this study demonstrates the therapeutic potential of acupuncture and lays the foundation for exploring its underlying mechanisms. Future research should focus on conducting high-quality, authoritative studies to strengthen the evidence and further elucidate the mechanisms of acupuncture.

The findings of this study underscore the significant therapeutic potential of acupuncture for SD-induced cognitive impairment and provide insights into its multi-target mechanisms.

## 5 Limitations

The present study has identified several methodological limitations that may affect the reliability of the results, as outlined below. (1) Regional and Language Bias: Although our search did not restrict geographic regions, the languages were limited to Chinese and English. This limitation may have led us to overlook a variety of non-English studies, such as those in Japanese or Korean. Acupuncture research is predominantly conducted in East Asia, and the included articles were only in Chinese and English. Consequently, all studies included in this research were conducted in China by Chinese researchers, which may introduce language bias. Additionally, the fact that some studies were not published in international journals increases the risk of publication bias. (2) Randomization and blinding: In this study, the five randomized controlled trials (RCTs) included did not explicitly report the specific method of randomized sequence generation and were evaluated as “unclear risk.” The lack of transparency in randomization methods may have affected the baseline balance between the experimental and control groups, thereby introducing selection bias. For example, systematic bias in assignment (e.g., nonrandomized assignment according to weight or behavioral characteristics) could exaggerate or underestimate the effect of an intervention. Although SYRCLE tools have tagged such risks, future studies should strictly follow animal experiment reporting guidelines, such as ARRIVE 2.0, and clearly describe the randomization process (e.g., computer-generated sequences or random number tables) to enhance confidence in the results (Percie du Sert et al., 2020). Six studies were not blinded to outcome assessors, which may have introduced detection bias. The outcome assessment of behavioral tests such as the Morris Water Maze (MWM) is subjective. If the assessor is aware of the animal group, it could unconsciously influence the data recording (for example, potentially more lenient timing for the treatment group). Although there are challenges in blinding animal experiments (such as the visibility of acupuncture operations), indirect blinding strategies are still recommended. These include having independent researchers perform behavioral tests and data analysis while concealing group information (Bebarta et al., 2003). Additionally, molecular marker detection methods such as ELISA and Western blot can reduce human bias through automated analysis. However, a strict blinded design is still required during the primary sample processing stage. (3) Limited number of studies: Despite an extensive search of major databases, only a limited number of studies met the inclusion criteria, which constrains the scope and generalizability of the findings. (4) Heterogeneity in Intervention Methods: Of the eight studies, two studies employed manual acupuncture (MA), while six utilized electroacupuncture (EA). EA is recognized for its effectiveness in treating persistent tissue damage (inflammation), nerve damage (neuropathic pain), cancer pain, and visceral pain. However, different EA frequencies and intensities activate the nervous system in distinct ways, thus playing different roles. For instance, high frequency (>50 Hz) is more likely to influence the serotonin (5-HT) system, while low frequency (2–4 Hz) is more likely to activate the opioid pathway (Zhang et al., 2014). The variation in EA frequencies used in the treatment of SD may result from different regulatory intensities and mechanisms of the BDNF/TrkB signaling pathway. Current research in this area is sparse, and further experiments are required to explore these specific effects. The effects exerted by different acupoint combinations also vary significantly. For example, the combination of GV20 with SP6 may enhance spatial memory by improving the function of the hippocampal-prefrontal circuit, whereas the impact of a single acupoint intervention may be limited (Liu et al., 2021). Additionally, the effects and cumulative impacts of different treatment cycles vary; short-cycle interventions (<7 days) may only alleviate acute oxidative stress, while long-cycle interventions (>14 days) are more conducive to the reconstruction of synaptic plasticity (MacPherson et al., 2010). However, the existing research cycles vary widely and require further standardization. Variations in acupoints, treatment durations, stimulation intensities, and frequencies across the studies contributed to high heterogeneity. This variability complicates the consistent evaluation of acupuncture’s efficacy. (5) Animal model limitations: The improved multiplatform water environment method induced chronic sleep deprivation by restricting REM sleep, while intraperitoneal injection of PCPA induced acute sleep deprivation by inhibiting serotonin synthesis. These two models may induce cognitive impairment through different mechanisms—chronic sleep fragmentation versus acute neurotransmitter imbalance—leading to variations in the targets of acupuncture intervention. The improved multiplatform water environment method more closely aligns with the pathological characteristics of human chronic sleep disorders, while the PCPA model is more suitable for studying the role of the monoaminergic system (Zielinski et al., 2016). This is one of the reasons why most of the articles included in this study adopted the modified multi-platform aquatic environment approach. Additionally, the use of three different animal types—Sprague–Dawley/Wistar and APP/PS1 transgenic mice—may still affect the heterogeneity of the study results. Differences such as smaller hippocampal volume and faster Aβ deposition in the mice could result in higher sensitivity to acupuncture intervention than in rats (Vorhees and Williams, 2014). Although the studies included in this research all used rodents, and no animal model can fully simulate all pathological features of the disease, many studies have shown high comparability in neurobehavioral assessments. Future studies should combine multiple models to comprehensively verify the multi-target effects of acupuncture. (6) Variability in Acupuncture Techniques: Differences in researchers’ skills may have introduced variability in outcomes. Variations in acupoint localization, needling techniques, insertion depth, and manipulation could impact treatment efficacy, leading to additional discrepancies in results.

## 6 Conclusion

This study demonstrates the potential of acupuncture, particularly EA and MA, in alleviating SD-induced cognitive impairment. The hypothesis that acupuncture can improve cognitive performance is supported by findings that it modulates key mechanisms such as BDNF–TrkB signaling, reduces oxidative stress, and regulates apoptosis. The efficacy of acupuncture is evidenced by improved outcomes in the Morris Water Maze and molecular evidence, including increased levels of BDNF, SOD, and GSH-Px, along with decreased MDA and Bax.

However, it should be noted that these results are based on preclinical animal studies, and their direct applicability to human conditions remains uncertain. Differences in physiology, treatment response, and the complexity of clinical presentations complicate the translation of these findings to human applications. Therefore, it is recommended that future research focus on bridging the gap between preclinical and clinical studies by integrating advanced methodologies such as functional imaging, molecular biomarker tracking, and precisely tailored acupuncture protocols for human use. Furthermore, long-term studies exploring the sustainability of acupuncture’s effects and its safety profile in human subjects are critical. This preliminary preclinical evidence underscores the potential of acupuncture as a multi-target intervention, paving the way for further clinical exploration and validation in managing cognitive impairments associated with SD.

## Data Availability

The original contributions presented in the study are included in the article/Supplementary material, further inquiries can be directed to the corresponding authors.
