# Supplementary material for: Effect of acupuncture on cognitive impairment induced by sleep deprivation in animal models: a preclinical systematic review and meta-analysis
Source: Front Aging Neurosci. 2025 Mar 19;17:1560032. doi: 10.3389/fnagi.2025.1560032 (PMC11962659; doi:10.3389/fnagi.2025.1560032)
Supplement: Supplementary file 1 [file Data_Sheet_1.docx]

**Table 1:** PubMed Search

| **NO** | **Search Details** | **Results** |
| --- | --- | --- |
| #1 | Search: "Acupuncture Therapy"[Mesh] Sort by: Most Recent | [30,331](https://pubmed.ncbi.nlm.nih.gov/?sort=date&term="Acupuncture+Therapy"[Mesh]) |
| #2 | Search: ("Acupuncture Therapy"[Mesh]) OR ((((((((Acupuncture Treatment) OR (Acupuncture Treatments))) OR (Therapy, Acupuncture)) OR (Pharmacoacupuncture Treatment)) OR (Treatment, Pharmacoacupuncture)) OR (Pharmacoacupuncture Therapy)) OR (Therapy, Pharmacoacupuncture) OR (Electroacupuncture)) | 40991 |
| #3 | #1 OR #2 | 40991 |
| #4 | Search: "Sleep Deprivation"[Mesh] Sort by: Most Recent | [11,410](https://pubmed.ncbi.nlm.nih.gov/?sort=date&term="Sleep+Deprivation"[Mesh]) |
| #5 | Search: (((((((((((((((((Deprivation, Sleep) OR (Insufficient Sleep Syndrome)) OR (Insufficient Sleep Syndromes)) OR (Syndrome, Insufficient Sleep)) OR (REM Sleep Deprivation)) OR (Deprivation, REM Sleep)) OR (Sleep Deprivation, REM)) OR (Sleep Fragmentation)) OR (Fragmentation, Sleep)) OR (Insufficient Sleep)) OR (Sleep, Insufficient)) OR (Inadequate Sleep)) OR (Sleep, Inadequate)) OR (Sleep Debt)) OR (Sleep Insufficiency)) OR (Insufficiencies, Sleep)) OR (Insufficiency, Sleep)) OR (Sleep Insufficiencies) | [26,861](https://pubmed.ncbi.nlm.nih.gov/?term=(((((((((((((((((Deprivation,+Sleep)+OR+(Insufficient+Sleep+Syndrome))+OR+(Insufficient+Sleep+Syndromes))+OR+(Syndrome,+Insufficient+Sleep))+OR+(REM+Sleep+Deprivation))+OR+(Deprivation,+REM+Sleep))+OR+(Sleep+Deprivation,+REM))+OR+(Sleep+Fragmentation))+OR+(Fragmentation,+Sleep))+OR+(Insufficient+Sleep))+OR+(Sleep,+Insufficient))+OR+(Inadequate+Sleep))+OR+(Sleep,+Inadequate))+OR+(Sleep+Debt))+OR+(Sleep+Insufficiency))+OR+(Insufficiencies,+Sleep))+OR+(Insufficiency,+Sleep))+OR+(Sleep+Insufficiencies)&sort=) |
| #6 | #4 OR #5 | [26,861](https://pubmed.ncbi.nlm.nih.gov/?term=("Sleep+Deprivation"[Mesh])+OR+((((((((((((((((((Deprivation,+Sleep)+OR+(Insufficient+Sleep+Syndrome))+OR+(Insufficient+Sleep+Syndromes))+OR+(Syndrome,+Insufficient+Sleep))+OR+(REM+Sleep+Deprivation))+OR+(Deprivation,+REM+Sleep))+OR+(Sleep+Deprivation,+REM))+OR+(Sleep+Fragmentation))+OR+(Fragmentation,+Sleep))+OR+(Insufficient+Sleep))+OR+(Sleep,+Insufficient))+OR+(Inadequate+Sleep))+OR+(Sleep,+Inadequate))+OR+(Sleep+Debt))+OR+(Sleep+Insufficiency))+OR+(Insufficiencies,+Sleep))+OR+(Insufficiency,+Sleep))+OR+(Sleep+Insufficiencies))&sort=) |
| #7 | Search: "Cognitive Dysfunction"[Mesh] Sort by: Most Recent | [40,909](https://pubmed.ncbi.nlm.nih.gov/?sort=date&term="Cognitive+Dysfunction"[Mesh]) |
| #8 | Search:(((((((((((((((((((((((Dysfunction,Cognitive) OR (Dysfunctions, Cognitive)) OR (Cognitive Disorder)) OR (Cognitive Disorders)) OR (Disorder, Cognitive)) OR (Disorders, Cognitive)) OR (Cognitive Impairments)) OR (Cognitive Impairment)) OR (Impairment, Cognitive)) OR (Impairments, Cognitive)) OR (Mild Cognitive Impairment)) OR (Cognitive Impairment, Mild)) OR (Cognitive Impairment, Mild)) OR (Impairment, Mild Cognitive)) OR (Impairments, Mild Cognitive)) OR (Mild Cognitive Impairments)) OR (Cognitive Decline)) OR (Cognitive Declines)) OR (Decline, Cognitive)) OR (Declines, Cognitive)) OR (Mental Deterioration)) OR (Deterioration, Mental)) OR (Deteriorations, Mental)) OR (Mental Deteriorations) | [329,904](https://pubmed.ncbi.nlm.nih.gov/?term=(((((((((((((((((((((((Dysfunction,+Cognitive)+OR+(Dysfunctions,+Cognitive))+OR+(Cognitive+Disorder))+OR+(Cognitive+Disorder))+OR+(Disorder,+Cognitive))+OR+(Disorders,+Cognitive))+OR+(Cognitive+Impairments))+OR+(Cognitive+Impairment))+OR+(Impairment,+Cognitive))+OR+(Impairments,+Cognitive))+OR+(Mild+Cognitive+Impairment))+OR+(Cognitive+Impairment,+Mild))+OR+(Cognitive+Impairment,+Mild))+OR+(Impairment,+Mild+Cognitive))+OR+(Impairments,+Mild+Cognitive))+OR+(Mild+Cognitive+Impairments))+OR+(Cognitive+Decline))+OR+(Cognitive+Declines))+OR+(Decline,+Cognitive))+OR+(Declines,+Cognitive))+OR+(Mental+Deterioration))+OR+(Deterioration,+Mental))+OR+(Deteriorations,+Mental))+OR+(Mental+Deteriorations)&sort=) |
| #9 | #7 OR #8 | [329,904](https://pubmed.ncbi.nlm.nih.gov/?term=(((((((((((((((((((((((Dysfunction,+Cognitive)+OR+(Dysfunctions,+Cognitive))+OR+(Cognitive+Disorder))+OR+(Cognitive+Disorder))+OR+(Disorder,+Cognitive))+OR+(Disorders,+Cognitive))+OR+(Cognitive+Impairments))+OR+(Cognitive+Impairment))+OR+(Impairment,+Cognitive))+OR+(Impairments,+Cognitive))+OR+(Mild+Cognitive+Impairment))+OR+(Cognitive+Impairment,+Mild))+OR+(Cognitive+Impairment,+Mild))+OR+(Impairment,+Mild+Cognitive))+OR+(Impairments,+Mild+Cognitive))+OR+(Mild+Cognitive+Impairments))+OR+(Cognitive+Decline))+OR+(Cognitive+Declines))+OR+(Decline,+Cognitive))+OR+(Declines,+Cognitive))+OR+(Mental+Deterioration))+OR+(Deterioration,+Mental))+OR+(Deteriorations,+Mental))+OR+(Mental+Deteriorations)&sort=) |
| #10 | #3 AND #6 AND #9 | 10 |

**Table 2:** EMbase Search

| **NO** | **Search Details** | **Results** |
| --- | --- | --- |
| #1 | 'acupuncture'/exp | 60037 |
| #2 | 'pharmacopuncture':ab,ti OR 'acupuncture treatment':ab,ti OR 'acupuncture treatments':ab,ti OR 'treatment, acupuncture':ab,ti OR 'therapy, acupuncture':ab,ti OR 'pharmacoacupuncture treatment':ab,ti OR 'treatment, pharmacoacupuncture':ab,ti OR 'pharmacoacupuncture therapy':ab,ti OR 'therapy, pharmacoacupuncture':ab,ti OR 'electroacupuncture':ab,ti | 14454 |
| #3 | #1 OR #2 | 60524 |
| #4 | 'sleep deprivation'/exp | 21887 |
| #5 | 'deprivation, sleep':ab,ti OR 'rem sleep deprivation':ab,ti OR 'deprivation, rem sleep':ab,ti OR 'sleep deprivation, rem':ab,ti OR 'sleep insufficiency':ab,ti OR 'insufficiencies, sleep':ab,ti OR 'insufficiency, sleep':ab,ti OR 'sleep insufficiencies':ab,ti OR 'insufficient sleep':ab,ti OR 'sleep, insufficient':ab,ti OR 'inadequate sleep':ab,ti OR 'sleep, inadequate':ab,ti OR 'sleep fragmentation':ab,ti OR 'fragmentation, sleep':ab,ti OR 'insufficient sleep syndrome':ab,ti OR 'insufficient sleep syndromes':ab,ti OR 'syndrome, insufficient sleep':ab,ti OR 'sleep debt':ab,ti | 9056 |
| #6 | #4 OR #5 | 28596 |
| #7 | 'cognitive defect'/exp | 650861 |
| #8 | 'cognitive dysfunctions':ab,ti OR 'dysfunction, cognitive':ab,ti OR 'dysfunctions, cognitive':ab,ti OR 'cognitive disorder':ab,ti OR 'cognitive disorders':ab,ti OR 'disorder, cognitive':ab,ti OR 'disorders, cognitive':ab,ti OR 'cognitive impairments':ab,ti OR 'cognitive impairment':ab,ti OR 'impairment, cognitive':ab,ti OR 'impairments,cognitive':ab,ti OR 'mild cognitiveimpairment':ab,ti OR 'cognitive impairment, mild':ab,ti OR 'cognitive impairments, mild':ab,ti OR 'aimpairment, mild cognitive':ab,ti OR 'cognitive decline':ab,ti OR 'cognitive declines':ab,ti OR 'impairments, mild cognitive':ab,ti OR 'mild cognitive impairments':ab,ti OR 'decline, cognitive':ab,ti OR 'declines, cognitive':ab,ti OR 'mental deterioration':ab,ti OR 'deterioration, mental':ab,ti OR 'deteriorations, mental':ab,ti OR 'mental deteriorations':ab,ti | 192231 |
| #9 | #6 OR #7 | 683,821 |
| #10 | #3 AND #6 AND #9 | 12 |

**Table 3:** Web of Science Search

| **NO** | **Search Details** | **Results** |
| --- | --- | --- |
| #1 | (((ALL=(Acupuncture Therapy)) OR ALL=(Acupuncture Treatments)) OR ALL=(Pharmacoacupuncture Treatment)) OR ALL=(Electroacupuncture) | [21,259](https://webofscience.clarivate.cn/wos/woscc/summary/c969a5c8-1cae-4616-bb20-064097520085-efdf55f0/relevance/1) |
| #2 | (((((((ALL=(Sleep Deprivation)) OR ALL=(Deprivation, Sleep)) OR ALL=(Insufficient Sleep Syndromes)) OR ALL=(REM Sleep Deprivation)) OR ALL=(Sleep Fragmentation)) OR ALL=(Inadequate Sleep)) OR ALL=(Sleep Debt)) OR ALL=(Sleep Insufficiencies) | 22803 |
| #3 | ((((((((((((((((((((((((ALL=(Cognitive Dysfunction)) OR ALL=(Dysfunction,Cognitive)) OR ALL=(Dysfunctions, Cognitive)) OR ALL=(Cognitive Disorder)) OR ALL=(Cognitive Disorders)) OR ALL=(Disorder, Cognitive)) OR ALL=(Disorders, Cognitive)) OR ALL=(Cognitive Impairments)) OR ALL=(Cognitive Impairment)) OR ALL=(Impairment, Cognitive)) OR ALL=(Impairments, Cognitive)) OR ALL=(Mild Cognitive Impairment)) OR ALL=(Cognitive Impairment, Mild)) OR ALL=(Cognitive Impairment, Mild)) OR ALL=(Impairment, Mild Cognitive)) OR ALL=(Impairments, Mild Cognitive)) OR ALL=(Mild Cognitive Impairments)) OR ALL=(Cognitive Declines)) OR ALL=(Decline, Cognitive)) OR ALL=(Declines, Cognitive)) OR ALL=(Mental Deterioration)) OR ALL=(Deterioration, Mental)) OR ALL=(Deteriorations, Mental)) OR ALL=(Mental Deteriorations)) | [315,302](https://webofscience.clarivate.cn/wos/woscc/summary/a5f812ae-df0d-46a2-8e57-e8889b6c180c-efded02e/relevance/1) |
| #4 | #1 AND #2 AND #3 | 8 |

**Table 4:** Cochrane Library Search

| **NO** | **Search Details** | **Results** |
| --- | --- | --- |
| #1 | MeSH descriptor: [Acupuncture Therapy] explode all trees | 7081 |
| #2 | (Acupuncture Treatment):ti,ab,kw OR(Acupuncture Treatments):ti,ab,kw OR(Therapy, Acupuncture):ti,ab,kw OR(Pharmacoacupuncture Treatment):ti,ab,kw OR(Treatment, Pharmacoacupuncture):ti,ab,kw OR(Pharmacoacupuncture Therapy):ti,ab,kw OR(Therapy, Pharmacoacupuncture):ti,ab,kw OR(Electroacupuncture):ti,ab,kw | 17809 |
| #3 | #1 OR #2 | 18157 |
| #4 | MeSH descriptor: [Sleep Deprivation] explode all trees | 1115 |
| #5 | (Deprivation, Sleep):ti,ab,kw OR(REM Sleep Deprivation):ti,ab,kw OR(Deprivation, REM Sleep):ti,ab,kw OR(Sleep Deprivation, REM):ti,ab,kw OR(Sleep Insufficiency):ti,ab,kw OR(Insufficiencies, Sleep):ti,ab,kw OR(Insufficiency, Sleep):ti,ab,kw OR(Sleep Insufficiencies):ti,ab,kw OR(Insufficient Sleep):ti,ab,kw OR(Sleep, Insufficient):ti,ab,kw OR(Inadequate Sleep):ti,ab,kw OR(Sleep, Inadequate):ti,ab,kw OR(Sleep Fragmentation):ti,ab,kw OR(Fragmentation, Sleep):ti,ab,kw OR(Insufficient Sleep Syndrome):ti,ab,kw OR(Insufficient Sleep Syndromes):ti,ab,kw OR(Syndrome, Insufficient Sleep):ti,ab,kw OR(Sleep Debt):ti,ab,kw | 4020 |
| #6 | #4 OR #5 | 4020 |
| #7 | MeSH descriptor: [Cognitive Dysfunction] explode all trees | 3996 |
| #8 | (cognitive dysfunctions):ti,ab,kw OR(dysfunction, cognitive):ti,ab,kw OR(dysfunctions, cognitive):ti,ab,kw OR(cognitive disorder):ti,ab,kw OR(cognitive disorders):ti,ab,kw OR(disorder, cognitive):ti,ab,kw OR(disorders, cognitive):ti,ab,kw OR(cognitive impairments):ti,ab,kw OR(cognitive impairment):ti,ab,kw OR(impairment, cognitive):ti,ab,kw OR(impairments,cognitive):ti,ab,kw OR(mild cognitiveimpairment):ti,ab,kw OR(cognitive impairment, mild):ti,ab,kw OR(cognitive impairments, mild):ti,ab,kw OR(aimpairment, mild cognitive):ti,ab,kw OR(cognitive decline):ti,ab,kw OR(cognitive declines):ti,ab,kw OR(impairments, mild cognitive):ti,ab,kw OR(mild cognitive impairments):ti,ab,kw OR(decline, cognitive):ti,ab,kw OR(declines, cognitive):ti,ab,kw OR(mental deterioration):ti,ab,kw OR(deterioration, mental):ti,ab,kw OR(deteriorations, mental):ti,ab,kw OR(mental deteriorations):ti,ab,kw | 56799 |
| #9 | #8 OR #9 | 56814 |
| #10 | #3 AND #6 AND #9 | 49 |

**Table 5:** CNKI Search

| **NO** | **Search Details** | **Results** |
| --- | --- | --- |
| #1 | 条件:（主题：针灸疗法）OR（主题：针刺疗法）OR（主题：电针）OR（主题：针药结合）AND（主题：睡眠剥夺模型）OR（主题：睡眠不足综合征）OR（主题：睡眠不足）OR（主题：REM睡眠剥夺）OR（主题：REM睡眠碎片化）OR（主题：睡眠障碍）AND（主题：认知功能障碍）OR（主题：认知障碍）OR（主题：轻度认知障碍）OR（主题：轻度神经认知障碍）OR（主题：精神衰退）OR（主题：认知损害） | 57 |

**Table 6:** Wanfang Search

| **NO** | **Search Details** | **Results** |
| --- | --- | --- |
| #1 | 主题:(针灸疗法 or 针刺疗法or 电针 or 针药结合) and 主题:(睡眠剥夺模型 or 睡眠不足综合征 or 睡眠不足 or REM睡眠剥夺 or REM睡眠碎片化 or 睡眠不足 or 睡眠障碍) and 主题:(认知功能障碍 or 轻度认知障碍 or 轻度神经认知障碍 or 认知减退 or 精神衰退 or 认知损害) | 15 |

**Table 7:** CBM Search

| **NO** | **Search Details** | **Results** |
| --- | --- | --- |
| #1 | ("针灸疗法"[常用字段:智能] OR "针刺疗法"[常用字段:智能] OR "电针"[常用字段:智能] OR "针药结合"[常用字段:智能]) AND ( 动物[特征词]) | [12418](javascript:historyLink('("%E9%92%88%E7%81%B8%E7%96%97%E6%B3%95"[%E5%B8%B8%E7%94%A8%E5%AD%97%E6%AE%B5:%E6%99%BA%E8%83%BD] OR "%E9%92%88%E5%88%BA%E7%96%97%E6%B3%95"[%E5%B8%B8%E7%94%A8%E5%AD%97%E6%AE%B5:%E6%99%BA%E8%83%BD] OR "%E7%94%B5%E9%92%88"[%E5%B8%B8%E7%94%A8%E5%AD%97%E6%AE%B5:%E6%99%BA%E8%83%BD] OR "%E9%92%88%E8%8D%AF%E7%BB%93%E5%90%88"[%E5%B8%B8%E7%94%A8%E5%AD%97%E6%AE%B5:%E6%99%BA%E8%83%BD]) AND ( %E5%8A%A8%E7%89%A9[%E7%89%B9%E5%BE%81%E8%AF%8D])')) |
| #2 | ("睡眠剥夺模型"[常用字段:智能] OR "睡眠不足综合征"[常用字段:智能] OR "睡眠不足"[常用字段:智能] OR "REM睡眠剥夺"[常用字段:智能] OR "REM睡眠碎片化"[常用字段:智能] OR "睡眠障碍"[常用字段:智能]) AND ( 动物[特征词]) | 1249 |
| #3 | ("认知功能障碍"[常用字段:智能] OR "认知障碍"[常用字段:智能] OR "轻度认知障碍"[常用字段:智能] OR "轻度神经认知障碍"[常用字段:智能] OR "精神衰退"[常用字段:智能] AND "认知损害"[常用字段:智能]) AND ( 动物[特征词]) | [2380](javascript:historyLink('("%E8%AE%A4%E7%9F%A5%E5%8A%9F%E8%83%BD%E9%9A%9C%E7%A2%8D"[%E5%B8%B8%E7%94%A8%E5%AD%97%E6%AE%B5:%E6%99%BA%E8%83%BD] OR "%E8%AE%A4%E7%9F%A5%E9%9A%9C%E7%A2%8D"[%E5%B8%B8%E7%94%A8%E5%AD%97%E6%AE%B5:%E6%99%BA%E8%83%BD] OR "%E8%BD%BB%E5%BA%A6%E8%AE%A4%E7%9F%A5%E9%9A%9C%E7%A2%8D"[%E5%B8%B8%E7%94%A8%E5%AD%97%E6%AE%B5:%E6%99%BA%E8%83%BD] OR "%E8%BD%BB%E5%BA%A6%E7%A5%9E%E7%BB%8F%E8%AE%A4%E7%9F%A5%E9%9A%9C%E7%A2%8D"[%E5%B8%B8%E7%94%A8%E5%AD%97%E6%AE%B5:%E6%99%BA%E8%83%BD] OR "%E7%B2%BE%E7%A5%9E%E8%A1%B0%E9%80%80"[%E5%B8%B8%E7%94%A8%E5%AD%97%E6%AE%B5:%E6%99%BA%E8%83%BD] AND "%E8%AE%A4%E7%9F%A5%E6%8D%9F%E5%AE%B3"[%E5%B8%B8%E7%94%A8%E5%AD%97%E6%AE%B5:%E6%99%BA%E8%83%BD]) AND ( %E5%8A%A8%E7%89%A9[%E7%89%B9%E5%BE%81%E8%AF%8D])')) |
| #4 | ((#1) AND (#2) AND (#3) AND ( 动物[特征词]) | 2 |

**Tble8：**维普网

| **NO** | **Search Details** | **Results** |
| --- | --- | --- |
| #1 | [(((((题名或关键词=针灸疗法 OR 题名或关键词=针刺疗法) OR 题名或关键词=电针) OR 题名或关键词=针药结合) AND ((((((题名或关键词=睡眠剥夺模型 OR 题名或关键词=睡眠不足综合征) OR 题名或关键词=睡眠不足) OR 题名或关键词=REM睡眠剥夺) OR 题名或关键词=REM睡眠碎片化) OR 题名或关键词=睡眠不足) OR 题名或关键词=睡眠障碍)) AND (((((题名或关键词=认知功能障碍 OR 题名或关键词=轻度认知障碍) OR 题名或关键词=轻度神经认知障碍) OR 题名或关键词=认知减退) OR 题名或关键词=精神衰退) OR 题名或关键词=认知损害))](https://qikan.cqvip.com/Qikan/search/index?LngMySearHistoryIdGuid=35537cd9-e2f4-4f6d-a368-f6d9f30fca25&from=Qikan_Article_History) | 2 |
