# Supplementary figures and images for: Effect of acupuncture on cognitive impairment induced by sleep deprivation in animal models: a preclinical systematic review and meta-analysis
Source: Front Aging Neurosci. 2025 Mar 19;17:1560032. doi: 10.3389/fnagi.2025.1560032 (PMC11962659; doi:10.3389/fnagi.2025.1560032)

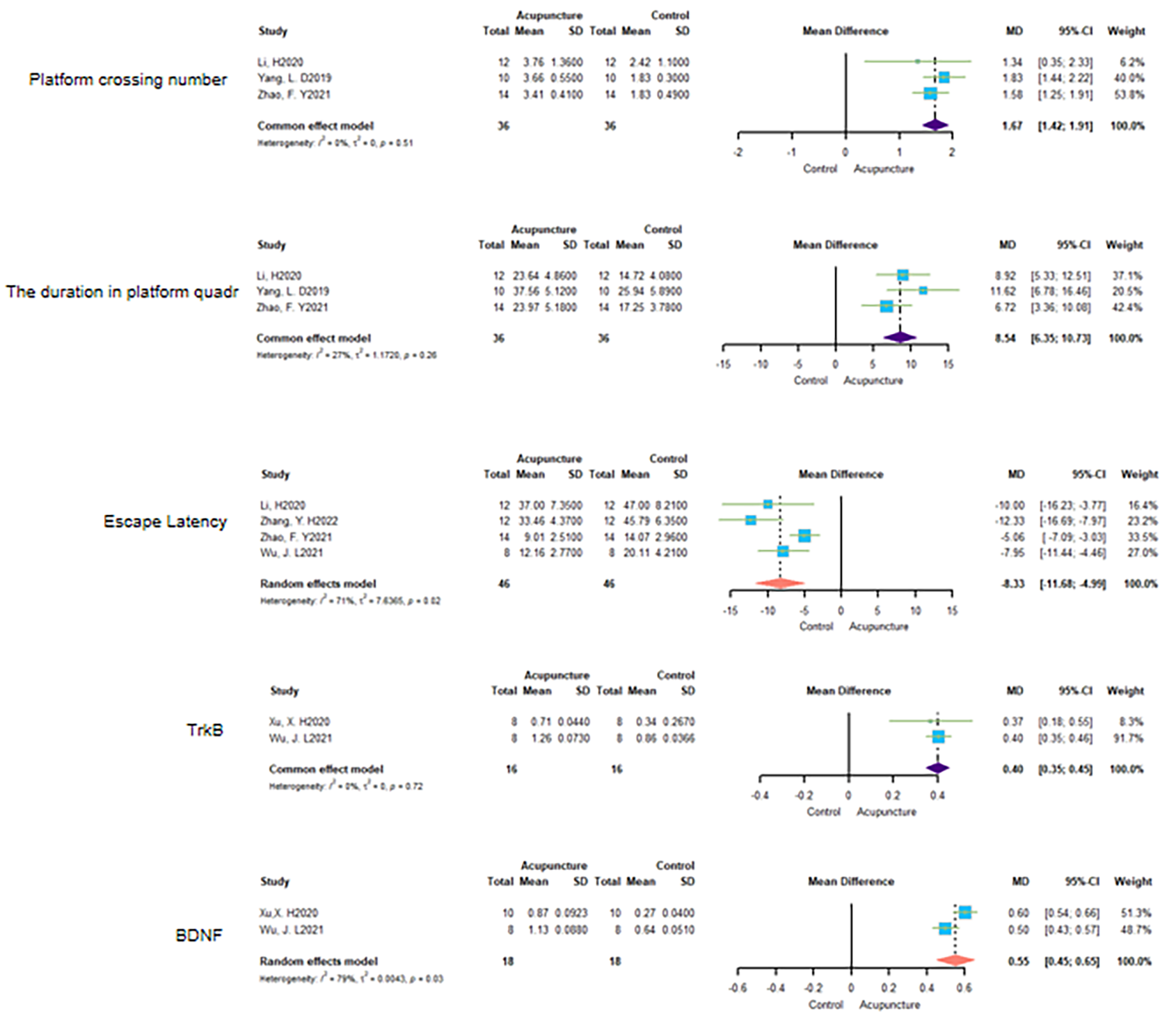

Supplement: Supplementary file 2 [file Image_1.tif]

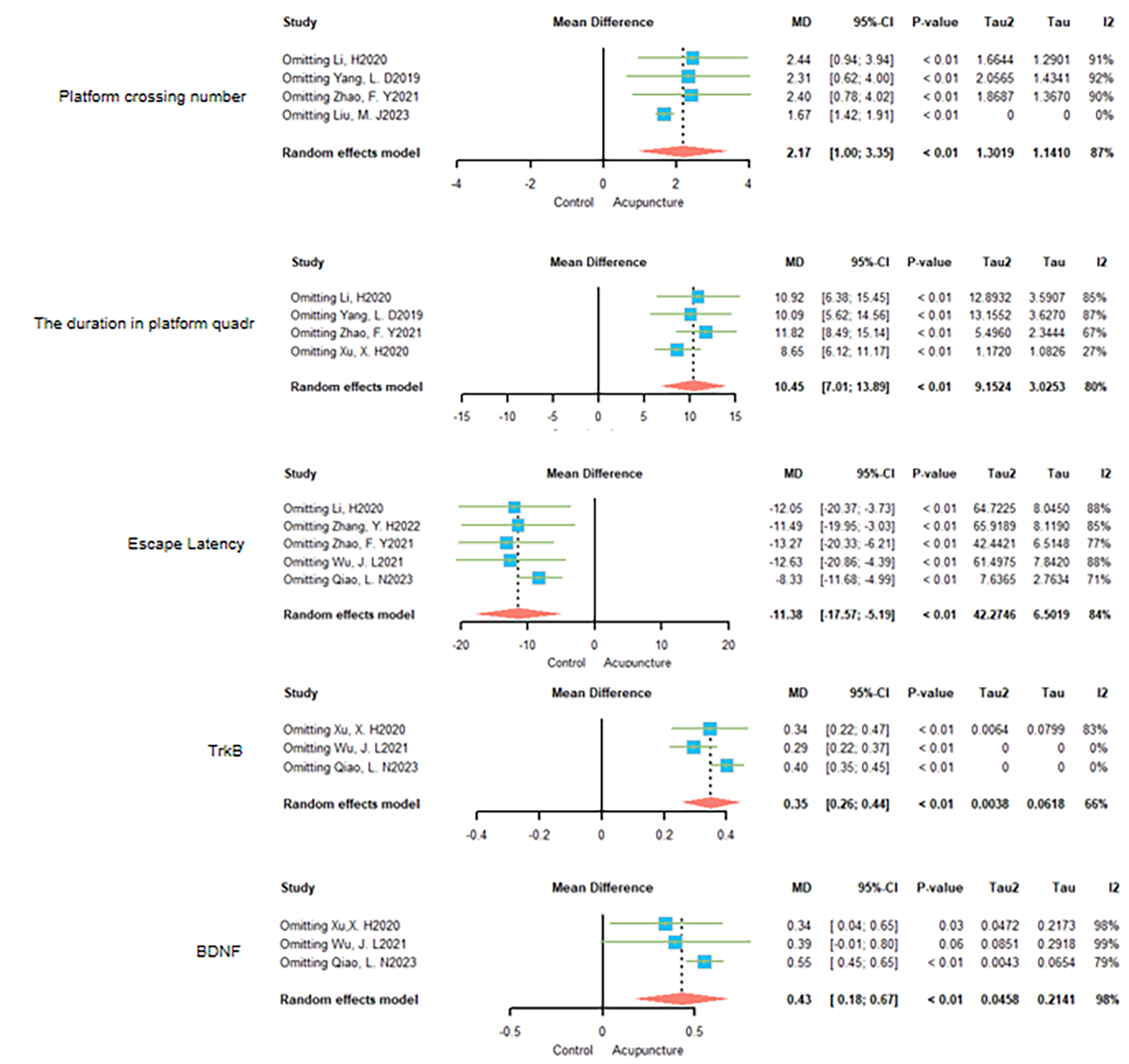

Supplement: Supplementary file 3 [file Image_2.tif]
